# Recombination with a vaccine strain drives the evolution of a more virulent phenotype in avian infectious bronchitis virus

**DOI:** 10.1016/j.psj.2026.106747

**Published:** 2026-03-08

**Authors:** Zhonglin Huang, Xu Wang, Hongfeng Ren, Pei Li, Yang Wang, Yafan Zhao, Jinchi Guo, Yingjie Chen, Pengran Wang, Xiaoran Li, Yanna Guo, Jianhui Li, Libin Liang, Junping Li

**Affiliations:** a Shanxi Key Laboratory of Animal Disease Research, Prevention and Control, College of Veterinary Medicine, Shanxi Agricultural University, Jinzhong 030801, China; b Laboratory of Poultry Production, College of Animal Science, Shanxi Agricultural University, Jinzhong 030801, China

**Keywords:** Avian infectious bronchitis virus, Phylogenetic analysis, Recombination, Virulence, Vaccine-driven evolution

## Abstract

Avian infectious bronchitis virus (IBV), a major pathogen in the poultry industry, evolves rapidly via genetic mutations and recombination. While recombination between field and live vaccine strains is well-documented, its direct role in viral virulence remains unclear. In this study, four novel IBV field strains (SX/2407, SX/2408, SX/2409, SX/2410) were isolated from a single layer farm in Shanxi Province, China, and genetically characterized. Comprehensive genomic analysis revealed that SX/2409 is a natural recombinant, with SX/2408 (co-isolated) as the major parent and a 4/91-like vaccine lineage as the minor parent. To assess the functional impact of this recombination, pathogenicity was compared in chicks. Notably, SX/2409 exhibited markedly enhanced virulence, with a significantly higher mortality rate (60%) than its major parent SX/2408 (20%). The other isolates, SX/2407 and SX/2410, caused 50% and 0% mortality, respectively. Our findings provide compelling field evidence that recombination with a live vaccine strain can directly drives the evolution of a more virulent IBV phenotype. These results highlight the potential evolutionary risks of current vaccination strategies and emphasize the critical need for robust molecular surveillance and development of safer next-generation vaccines.

## Introduction

Avian infectious bronchitis (IB), caused by the avian infectious bronchitis virus (IBV), is an acute, highly contagious disease affecting chickens (Keep, et al., 2022). The virus primarily targets the respiratory, urinary, and reproductive tracts, leading to reduced body weight and feed conversion efficiency in broilers, and decreased egg production and quality in layers, resulting in substantial economic losses (Bo, et al., 2022). IBV is highly infectious with an incubation period of 24-48 hours and can infect chickens of all ages. Infected birds typically exhibit a range of respiratory symptoms such as sneezing, coughing, and tracheal rales. Infection with nephropathogenic IBV strains can cause high mortality, characterized by urate deposition in renal tubules, giving the kidneys a mottled, pale, and swollen appearance, known as "mottled kidney" (Hassan, et al., 2021). Infections in young chicks by many IBV strains can cause permanent damage to the oviduct, leading to significantly reduced egg production and quality upon sexual maturity (Amarasinghe, et al., 2018). IBV infection often damages the tracheal epithelium, predisposing chickens to secondary infections by other pathogens such as H9N2 avian influenza virus, Mycoplasma gallisepticum, and Escherichia coli, thereby increasing mortality (Liang, et al., 2024; Yang, et al., 2024). IBV is prevalent worldwide, its high mutation rate, driven by environmental pressure and vaccine immunity, results in numerous serotypes, making research and control of IBV remain critically important (Chen, et al., 2019; Cheng, et al., 2019; Li, et al., 2019).

IBV belongs to the order *Nidovirales*, family *Coronaviridae*, genus *Gammacoronavirus*. The IBV virion is composed of four structural proteins: the spike (S) glycoprotein, membrane (M) protein, envelope (E) protein, and nucleocapsid (N) protein (Liu, et al., 2019; Yin, et al., 2016; Zhao, et al., 2019). The S protein, located on the outermost layer of the virion, forms the characteristic club-shaped projections on the viral envelope. With a molecular weight ranging from 150 to 200 kDa, it is the largest structural protein and plays a crucial role in viral infectivity, tissue tropism, virulence, and pathogenicity (da Silva, et al., 2022; Du, et al., 2022; Zhu, et al., 2021). Serotype is determined by neutralizing antibodies targeting antigenic epitopes on the S protein, which are closely associated with hypervariable regions (HVRs) within the S1 gene subunit. Unlike the relatively conserved N gene, the S1 gene is prone to mutation. The weak cross-protection between different serotypes, largely due to S1 variability, contributes to the vast number of IBV serotypes. Currently, the main prevalent serotypes in China include QX type (GI-19), GVI-1, 793/B (GI-13), and TW-1 (GI-7) (Lian, et al., 2021; Mendoza-González, et al., 2022). Internationally, IBV is classified into 10 genotypes and 41 lineages. Among these, the GI genotype comprises 30 lineages (GI-1 to GI-30), GVII and GVIII-1 each include 2 lineages, while the remaining 7 genotypes each contain only one lineage (Bali, et al., 2021; Bouwman, et al., 2020).

The high stocking densities common in modern poultry farming create favorable conditions for viral replication, maintenance, and transmission, facilitating the emergence of novel viruses and viral populations (Hou, et al., 2020). Studies have found that suboptimal levels of neutralizing antibodies in chickens have been demonstrated to promote viral entry into host cells and enhance viral replication efficiency (Reynolds, et al., 2024). When antibody titers in immunized flocks drop to such suboptimal levels, and combined with the large reservoir of susceptible hosts associated with intensive poultry farming and inadequate housing ventilation, these coexisting factors collectively elevate the challenges of preventing and controlling major avian viral diseases including avian influenza, infectious bronchitis, and Newcastle disease (Li, et al., 2025; Masoud, et al., 2024). Recombination between different field strains during natural mixed infections, coupled with the widespread use of heterologous IBV vaccines in the field, plays a significant role in the emergence of new IBV genetic variants (Fan, et al., 2019). Among 32 IBV strains isolated from Southwest China between 2018 and 2020, 8 exhibited recombination. Comparative evaluation of the protective efficacy of four attenuated vaccines against these strains revealed that even the most effective one, LDT3-A, could not completely prevent infection by field strains (Fan, et al., 2019; Lian, et al., 2021; Zhou, et al., 2018). Ma and colleagues found that six IBV strains they isolated originated from recombination events between the 4/91 vaccine and GI-19 strains, and the commonly used 4/91 vaccine could not provide sufficient protection against the predominantly circulating GI-19 lineage in China (Ma, et al., 2019; Nam, et al., 2019). In addition, it has been reported that coronaviruses, including IBV, can undergo mutation and recombination due to the error-prone nature of their RNA-dependent RNA polymerase, which lacks proofreading ability, especially when different lineages co-exist in a biological sample (Nam, et al., 2019; Xu, et al., 2018).

In this study, four IBV strains of different genotypes were simultaneously isolated and identified in 2024 from a large-scale layer farm in North China that practiced routine IBV vaccination. To analyze their biological and molecular genetic characteristics, whole-genome sequencing and bioinformatics analyses were performed on these four strains, elucidating their origins and potential recombination events. We also evaluated the pathogenicity of the four IBV strains in chickens, revealing significantly different pathogenicities with mortality rates ranging from 0% to 60% in specific pathogen-free (SPF) chickens. The analysis of recombination and pathogenicity among different IBV strains within the same farm provides important data to support understanding the genetic evolution of IBV.

## Materials and methods

### Virus isolation and purification

In July 2024, tissue samples and swabs were collected from suspected diseased chickens at an intensive layer farm in Changzhi, Shanxi Province. Four IBV strains were identified using IBV-specific detection primers. Positive swab or tissue samples were inoculated into the allantoic cavity of 9-day-old specific pathogen-free (SPF) chicken embryos. The viruses were purified through three consecutive passages, and the four isolated strains were named CK/CH/SX/2407, CK/CH/SX/2408, CK/CH/SX/2409, and CK/CH/SX/2410 (hereinafter referred to as SX/2407, SX/2408, SX/2409, SX/2410). The 50% embryo infectious dose (EID₅₀) for each strain was calculated using the Reed-Muench method.

### SPF chicks and embryos

The SPF chicken embryos and SPF chicks used in this study were purchased from Beijing Boehringer Ingelheim Merial Vital Laboratory Animal Technology Co., Ltd. (Beijing, China). All SPF embryos were incubated at 37°C and 65% relative humidity until they reached 9 days of age. All SPF chicks were housed in isolators under identical conditions and provided with the same feed and water throughout the experiment.

### Sequence analysis

Viral total RNA was extracted from allantoic fluid using the chloroform extraction method. Reverse transcription was performed according to the manufacturer's instructions of the Tolo Script All-in-one RT Easy Mix for qPCR kit (TOLOBIO, Shanghai, China). The S1 gene was amplified using specific primers as described previously. The amplification products were analyzed by 1% agarose gel electrophoresis. A band of approximately 1700 bp corresponding to the S1 gene was excised and purified using the E.Z.N.A.® Gel Extraction Kit (Omega Bio-Tek). Sequencing was performed by Sangon Biotech Co., Ltd (Shanghai, China). The S1 gene sequences of the four IBV isolates were assembled using the SeqMan software in the DNASTAR package. Eighty-five reference S1 gene sequences representing 9 genotypes and 37 lineages were downloaded from the GenBank database. The assembled S1 sequences of the four isolates were aligned with these references. Multiple sequence alignment was performed using MAFFT v7.526. A maximum likelihood phylogenetic tree based on the S1 gene was constructed using IQ-TREE software with the Single-Locus Tree analysis method and 1000 bootstrap replicates. The resulting tree was visualized using the ChiPlot website (https://www.chiplot.online/).

### Viral RNA sequencing and genome assembly

The complete RNA genomes of the four IBV strains were subjected to high-throughput sequencing at Sangon Biotech Co., Ltd (Shanghai, China). Briefly, viral RNA samples were randomly fragmented using divalent cations in Fragmentation Buffer. The fragmented RNA was used as template to synthesize double-stranded cDNA with random oligonucleotides as primers, constructing the sequencing library. During sequencing on the flow cell, four fluorescently labeled dNTPs, DNA polymerase, and adapter primers were added for amplification. As each complementary strand extended within a sequencing cluster, the incorporation of a fluorescently labeled dNTP released a corresponding fluorescent signal. The sequencer captured these signals, and computer software converted the optical signals into sequencing peaks to obtain sequence information. Clean reads were then assembled de novo, and contigs potentially originating from the viral genome were screened based on a provided reference sequence (CK/CH/SX/2204, OQ189491). The complete genome sequences, each approximately 27.6 kb in length, were assembled for each strain (SX2407, SX2408, SX2409, and SX2410) and subsequently deposited in GenBank under accession numbers PX759540, PX759541, PX759542, and PX759543.

### Recombination analysis

To further analyze potential recombination events in the genomes of the four IBV strains, 49 complete IBV genomes, including classical vaccine strains and strains from various genotypes, were downloaded from NCBI. The complete genomes of the four isolates were aligned with the 49 reference sequences using MAFFT. The recombination analysis software RDP4 was applied to infer recombination events. RDP4 incorporates seven recombination detection methods: RDP, GENECONV, BootScan, MaxChi, Chimaera, SiScan, and 3Seq (Wang, et al., 2022). Parameters for each method were set according to the RDP4 manual, with an acceptable P-value threshold of 0.05. An event was considered a credible recombination if it was detected by at least five of the seven methods with a P-value ≤ 10⁻¹². To validate the results, credible recombination events inferred by RDP4 were re-analyzed using the BootScan method in SimPlot software to verify the recombination relationships between the recombinant strain, major parent, and minor parent. A window size of 500 nucleotides and a step size of 20 nucleotides were used.

### Pathogenicity evaluation of the IBV strains

A total of 113 one-day-old SPF chicks were randomly divided into five groups: A, B, C, D, and E. Groups A, B, C, and D each contained 22 chicks, with 10 assigned to clinical observation and the remaining 12 allocated for organ viral load detection and histopathological examination. Groups A, B, C, and D were inoculated via the ocular and nasal routes (eye-drop and intranasal) with strains SX/2407, SX/2408, SX/2409, and SX/2410, respectively, at a dose of 10⁵ EID₅₀ per chick in 0.2 ml. Group E (10 chicks) served as the negative control, receiving an equivalent volume of phosphate-buffered saline (PBS). Fifteen additional SPF chicks were introduced on day 1 post-inoculation (3 chicks randomly placed into each of groups A, B, C, D, and E) to monitor contact transmission. All chicks were individually marked. For the clinical observation groups (n=10 per virus group), chicks were weighed daily for 14 days, and clinical signs were recorded. From groups A, B, C, and D, three inoculated chicks and the three contact chicks in each group were sampled at 3, 5, 7, and 9 days post-inoculation (dpi). Oropharyngeal and cloacal swabs were collected and placed in 2 ml EP tubes containing 1 ml of sterile PBS for viral shedding detection. At 3, 5, 7, and 9 dpi, three chicks from groups A, B, C, and D were randomly selected and euthanized. Tracheas were collected for ciliary activity assessment. Tissue samples including lung, kidney, liver, and spleen were collected. A portion of tissues with visible lesions was fixed in 4% paraformaldehyde for histological examination. The remaining tissues were placed in sterile PBS containing antibiotics and stored at -80°C for viral RNA load quantification by reverse transcription quantitative PCR (RT-qPCR).

### Clinical signs and tracheal ciliary activity scoring

To assess clinical morbidity in the observation groups, chicks were monitored daily post-challenge. Clinical signs including feather condition, posture, mental status, respiratory signs, diarrhea, and mortality were recorded and scored. A clinical score was assigned: 0 (healthy), 1 (mild symptoms), 2 (moderate symptoms), 3 (severe symptoms), and 4 (death) (Xu, et al., 2022; Yang, et al., 2024). For tracheal ciliary activity assessment, the trachea was divided into upper, middle, and lower sections. Tracheal rings approximately 3 mm thick were cut, rinsed thoroughly 5-8 times with sterile PBS, and placed ring-face up in a 96-well plate, with each well containing 200 µL of MEM supplemented with 10% fetal bovine serum (FBS). Ciliary activity was observed under an inverted microscope at 400x magnification and scored: 0 (100% ciliary movement), 1 (75%-100% movement), 2 (50%-75% movement), 3 (25%-50% movement), and 4 (0-25% movement) (Oade, et al., 2019).

### Histopathology

Tissue specimens from trachea, lung, and kidney were fixed in 4% formaldehyde for 48 hours at room temperature, embedded in paraffin, and sectioned at 5 µm thickness. Sections were stained with hematoxylin and eosin (H&E) and examined under a light microscope at 400x magnification for pathological changes.

### Organ viral load assay

Total RNA was extracted from collected tissues (trachea, lung, kidney, liver, spleen) and swabs (oropharyngeal, cloacal) according to the viral RNA extraction kit instructions. A pair of specific primers, namely IBV-TY-F (5′-CCTGGAAACGAACGGTAGACC-3′) and IBV-TY-R (5′-TAGTGGGCGTCCTAGTGCTG-3′), as well as a specific TaqMan probe IBV-TY-P (5′-FAM-ATCGTACTCCGCGTGGCCCCG-TAMRA-3′), were synthesized targeting the conserved region of the 3′ untranslated region (3′UTR). All primers and the probe were commercially synthesized by Beijing Tsingke Biotech Co., Ltd. The RT-qPCR reaction mixture consisted of 10 µL of 2 × One Step RT-qPCR Buffer II, 0.4 µL of Pro Taq HS DNA Polymerase (5 U/µL), 0.4 µL of Evo M-MLV RTase Enzyme Mix II, 0.6 µL each of forward and reverse primers, 0.8 µL of probe, 2 µL of RNA template, and RNase-free water to a final volume of 20 µL. The thermal cycling conditions were: 42°C for 5 min; 95°C for 30 s; followed by 40 cycles of 95°C for 5 s and 56°C for 30 s.

### Statistical analysis

Statistical analysis was performed using GraphPad Prism version 8 for Windows. Student's t-test was used to determine whether there were significant differences in the data between the different groups. A P-value < 0.05 was considered statistically significant (denoted as a, P < 0.05; b, P < 0.01; c, P < 0.001).

## Results

### IBV isolation, identification, and EID₅₀ determination

**Fig. 1:**
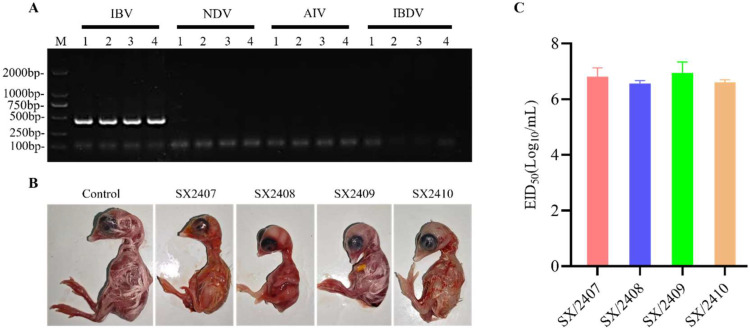
**Identification of four IBV isolates.** (A) Tissue and swab samples were inoculated into the allantoic cavity of 9-day-old SPF embryonated chicken eggs. After 3 days, the allantoic fluid was collected and tested by RT-PCR for Infectious Bronchitis Virus (IBV), Newcastle Disease Virus (NDV), Avian Influenza Virus (AIV), and Infectious Bursal Disease Virus (IBDV). Lanes 1, 2, 3, and 4 correspond to isolates SX/2407, SX/2408, SX/2409, and SX/2410, respectively. (B) The four isolates were inoculated into the allantoic cavity of 9-day-old SPF embryonated chicken eggs. Embryonic lesions, such as dwarfing or malformation, were observed 6 days post-inoculation. (C) Allantoic fluid containing each isolate was serially tenfold diluted in PBS and inoculated into the allantoic cavities of 9-day-old SPF embryonated chicken eggs. The 50% Embryo Infective Dose (EID₅₀) was calculated 6 days later using the Reed-Muench method.

In July 2024, four IBV strains were successfully identified from suspected cases at an intensive layer farm in Shanxi, which practiced routine vaccination with attenuated IBV live vaccines (Fig. 1A). The strains were named SX/2407, SX/2408, SX/2409, SX/2410. To study their biological characteristics, SX/2407, SX/2408, SX/2409, and SX/2410 were passaged three times in SPF embryos. All inoculated embryos exhibited typical lesions such as stunted growth or dwarfism (Fig. 1B). Allantoic fluid from the third passage was serially diluted (10⁻¹ to 10⁻⁸) and inoculated into embryos. After 144 hours of incubation, the EID₅₀ for each strain was calculated using the Reed-Muench method. The titers were: SX/2407, 10⁶·⁸¹ EID₅₀/ml; SX/2408, 10⁶·⁵⁶ EID₅₀/ml; SX/2409, 10⁶·⁹⁴ EID₅₀/ml; and SX/2410, 10⁶·⁶ EID₅₀/ml (Fig. 1C).

### Genetic evolution analysis of IBV isolates

To determine the genotype of the four IBV strains, the S1 gene (approximately 1742 bp) was amplified using specific primers and sequenced as described previously (Yang, et al., 2024). The obtained S1 sequences were assembled using SeqMan software and subjected to BLAST analysis against the NCBI database. Results showed that SX/2407 shared the highest homology (98.58%) with strain TW2575/98 (GenBank: DQ646405) isolated from Taiwan, China. SX/2408 shared 98.15% homology with strain CK/CH/JS/CZ200515 (GenBank: PP438798) from Jiangsu, China. SX/2410 shared 99.76% homology with strain CK/CH/SX/2204 (GenBank: OQ189491) previously isolated in Shanxi by our laboratory. SX/2409 shared 97.89% homology with strain ck/CH/LAH/08I (GenBank: PP987313) which was isolated from China in 2008.

**Fig. 2:**
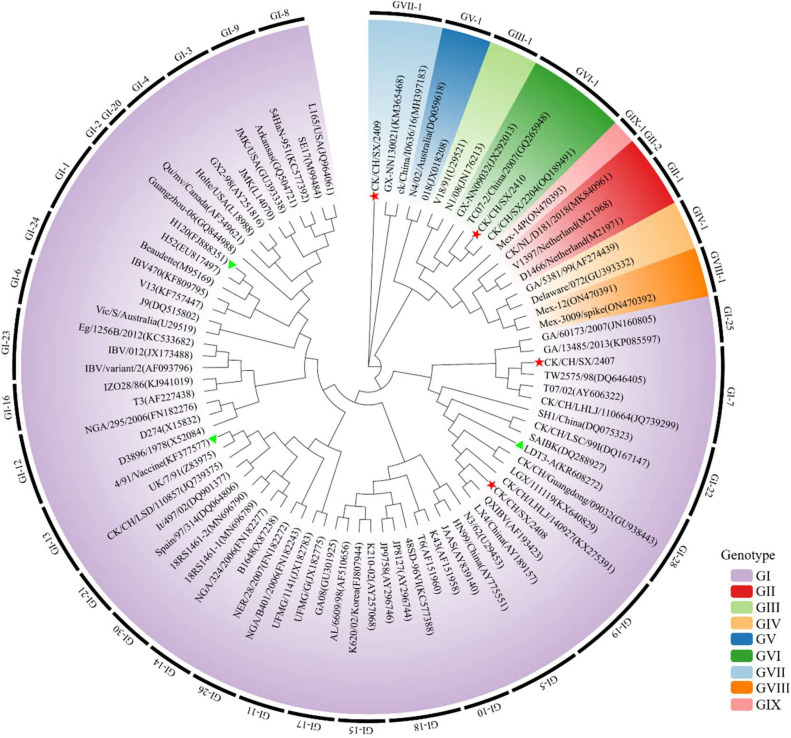
**Phylogenetic analysis of four IBV isolates from North China.** A phylogenetic tree was constructed for isolates SX/2407, SX/2408, SX/2409, and SX/2410 using 85 S1 gene sequences representing different genotypes, obtained from NCBI as reference sequences. The tree was generated using the maximum likelihood method in IQ-TREE with 1, 000 bootstrap replicates and subsequently visualized using ChiPlot (https://www.chiplot.online/). The IBV isolates obtained in this study are marked with red pentagrams, while vaccine strains are indicated by green triangles.

The S1 gene sequences of the four isolates were aligned with 85 reference sequences representing 9 genotypes and 37 lineages. The phylogenetic tree revealed that SX/2407 clustered within the GI-7 lineage, SX/2408 within the GI-19 lineage, SX/2409 within the GVII-1 lineage, and SX/2410 within the GVI-1 lineage, closely related to the typical GVI-1 strain TC0-2 (Fig. 2).

### Recombination analysis

**Fig. 3:**
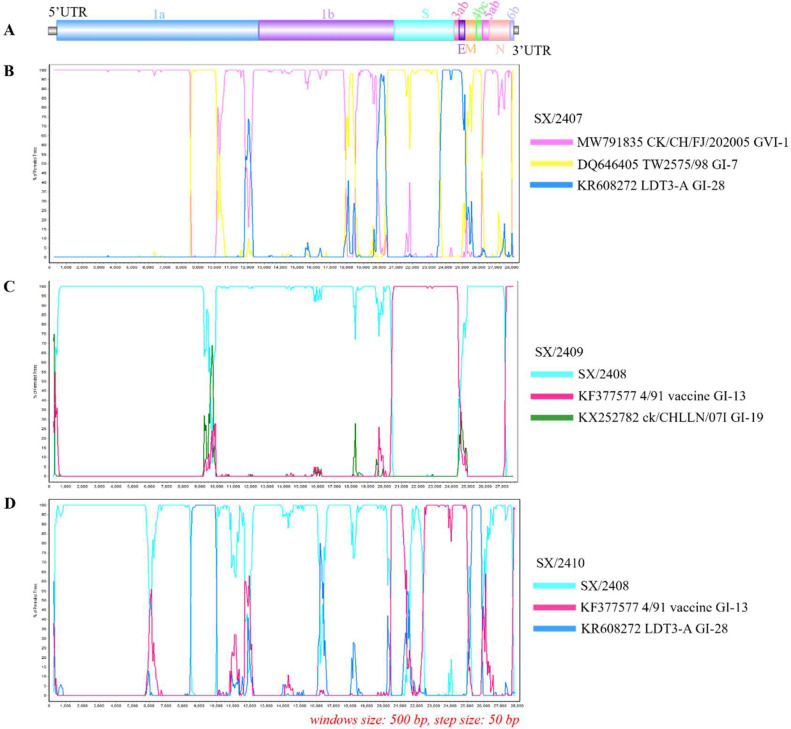
**Genomic characterization and recombination analysis of IBV.** (A) Schematic diagram of the IBV genome. (B-D) Recombination analysis of the complete genome sequences of SX/2407 (B), SX/2409 (C), and SX/2410 (D) was performed using BootScan. The analysis was conducted with SimPlot v3.5.1, using a window size of 500 bp and a step size of 50 bp. The identified recombination sites correspond to the full genome map in (A).

Given that all four IBV strains were isolated from the same laying hen farm practicing routine vaccination with attenuated live vaccines, it was hypothesized that these strains might have acquired immune evasion capabilities through genetic recombination. Nucleic acids were extracted from the four strains for high-throughput sequencing, yielding their complete genome sequences. The sequences were aligned with 49 reference complete genomes using MAFFT. RDP4 was used to infer recombination events, and SimPlot validation confirmed events consistent with RDP4 findings. Results indicated that SX/2407, SX/2409, and SX/2410 were recombinant, with SX/2409 and SX/2410 sharing SX/2408 as their major parental strain (Fig. 3). No recombination event was detected for SX/2408.

The SX/2407 genome originated from three lineages. BootScan analysis revealed at least four recombination breakpoints in the SX/2407 genome. CK/CH/FJ/202005 (MW791835, GVI-1) served as the major parent, while TW2575/98 (DQ646405, GI-7) and LDT3-A (KR608272, GI-28) were minor parents. Major recombination fragments in SX/2407 spanned nucleotides 8600-10100, 20500-23500, and 25450-26150, covering parts of the 1a, S, 4bc, and 5ab genes (Fig. 3B). A recombination fragment with LDT3-A spanned nucleotides 23750-25150, covering the 3ab, E, and M regions.

The SX/2409 genome originated from the GI-19 and GI-13 lineages. It resulted from recombination between the major parent SX/2408 (GI-19) and the minor parent 4/91 vaccine (KF377577, GI-13). BootScan analysis indicated a major recombination breakpoint in SX/2409 spanning nucleotides 20450-24350, covering the S, 3ab, and part of the E gene (Fig. 3C).

The SX/2410 genome resulted from recombination involving three lineages: GI-19, GI-13, and GI-28. The major parent was SX/2408 (GI-19), and the minor parent was the 4/91 vaccine (KF377577, GI-13). BootScan showed major recombination breakpoints at nucleotides 20450-21300 and 22400-25000, covering part of the S gene and the complete 3ab, E, and M genes. Additionally, nucleotides 8550-9950 and 25200-25850 in the SX/2410 genome were likely derived from LDT3-A (KR608272, GI-28), covering parts of the 1a and 5ab genes (Fig. 3D).

### Clinical signs in infected chickens

**Fig. 4:**
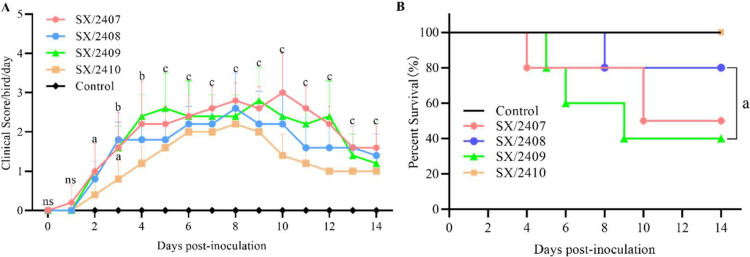
**Clinical symptom scores and survival curves of chickens following infection with specific IBV strains.** (A) Clinical scores of chickens infected with SX/2407, SX/2408, SX/2409, and SX/2410. Chickens inoculated with PBS served as the control group (n=10 per group). Only show significance between the clinical observation group and the control group. Statistical significance is indicated as follows: NS, not significant; a, P<0.05; b, P<0.01; c, p < 0.001. (B) Survival rates of 1-day-old SPF chickens infected with SX/2407, SX/2408, SX/2409, SX/2410, or PBS (control). Mortality was monitored for an observation period of 14 days. Only show the comparison results of SX/2408 and SX/2409. Statistical significance is indicated as follows: a, P<0.05.

To evaluate the pathogenicity of the four IBV strains, one-day-old SPF chicks were inoculated via the ocular and nasal routes with a dose of 10⁵ EID₅₀ per chick (0.2 ml). A PBS-inoculated group served as the negative control. Clinical signs in the observation groups (n=10 per group) were recorded and scored daily for 14 days post-inoculation. Chicks infected with SX/2407 and SX/2409 began to exhibit apparent clinical signs at 3 dpi, including neck extension, gasping, depression, lethargy, and in severe cases, paralysis. Chicks infected with SX/2408 and SX/2410 exhibited gasping, sneezing, and diarrhea at 3 dpi, with yellowish-brown watery feces and vent prolapse observed (Fig. 4A). Body weight gain in all infected groups was lower and slower compared to the control group, with significant differences noted at 5, 7, and 14 dpi (Fig. S1). During the observation period, SX/2407 infection resulted in 5 deaths between 4-10 dpi (50% mortality); SX/2409 infection caused 6 deaths between 5-10 dpi (60% mortality); SX/2408 infection led to 2 deaths at 8 dpi (20% mortality) (Fig. 4B). No mortality occurred in the SX/2410 or PBS control groups (Fig. 4B).

### Necropsy and gross pathological observations

**Fig. 5:**
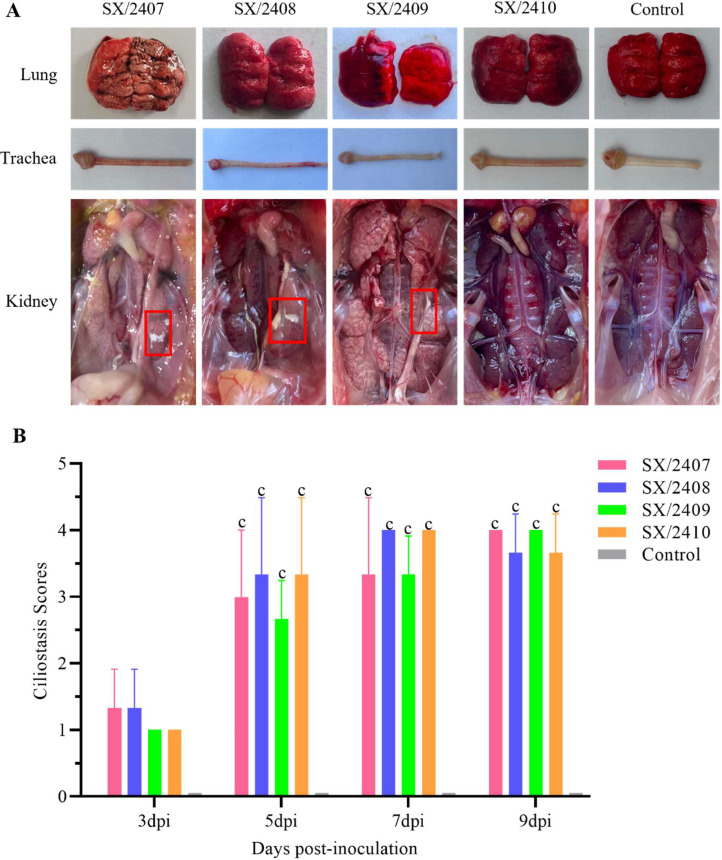
**Gross lesions in tissues and tracheal ciliary activity scores of chickens infected with the four IBV strains.** (A) In comparison to the PBS control group, chickens infected with SX/2407, SX/2408, SX/2409, and SX/2410 exhibited visible lesions in the lungs, trachea, and kidneys. (B) Assessment of tracheal ciliary activity in infected chickens at 3, 5, 7, and 9 days post-infection. Statistical significance is indicated as follows: c, p < 0.001.

Necropsy findings revealed small amounts of yellow mucus in the trachea of chicks infected with SX/2407 and SX/2409. Kidneys from chicks infected with SX/2407, SX/2408, and SX/2409 were swollen with evident urate deposition, and SX/2409 induced more severe renal lesions than its parental strain SX/2408. Lung tissues showed necrosis, hemorrhage, and congestion. Pathological changes induced by SX/2410 were milder. No lesions were observed in any tissues from the PBS control group (Fig. 5A).

Tracheal ciliary activity was assessed at 3, 5, 7, and 9 dpi. Ciliary activity began to decrease at 3 dpi in all infected groups. The highest average ciliostasis scores (4.0) were observed at 7 dpi for SX/2408 and SX/2410, and at 9 dpi for SX/2407 and SX/2409 (Fig. 5B). Ciliostasis scores in all infected groups were significantly higher than in the control group, which consistently scored 0.0 throughout the observation period.

### Histopathological lesion analysis

**Fig. 6:**
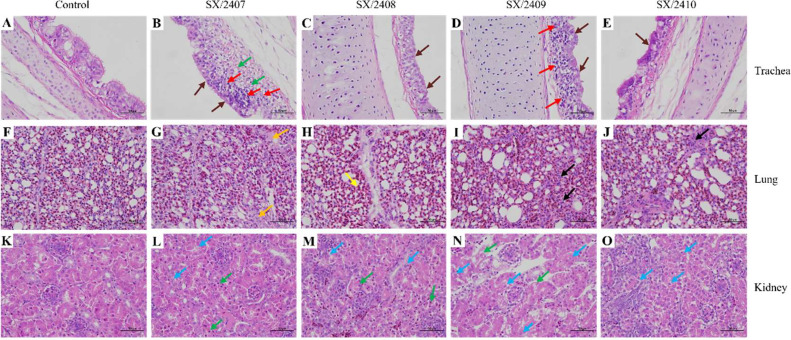
**Histopathological changes in SPF chickens infected with the four IBV isolates.** Tissue sections from the trachea (A-E), lungs (F-J), and kidneys (K-O) were fixed in 4% paraformaldehyde (PFA) and stained with hematoxylin and eosin (H&E) for pathological examination. Changes are indicated by colored arrows: loss and lodging of cilia (brown arrows), necrosis of epithelial cells (green arrows), lymphocyte infiltration (red arrows), focal indistinct structure of respiratory capillaries (black arrows), focal loose structure of respiratory capillaries (orange arrows), capillary hemorrhage (yellow arrows), and edema of renal tubular epithelium (blue arrows). No significant lesions were observed in the trachea (A), lungs (F), or kidneys (K) of the control group.

Histopathological examination confirmed that SX/2407 and SX/2409 caused severe damage to the trachea, lungs, and kidneys, while lesions induced by SX/2408 and SX/2410 were comparatively milder (Fig. 6). Tracheal lesions included cilia loss or disarray, thickening of the lamina propria, irregular arrangement of mucosal epithelial cells, focal epithelial necrosis with nuclear fragmentation, and lymphocyte infiltration. Lung lesions included focal loss of normal respiratory capillary structure, loosening of alveolar septa, and capillary hemorrhage. Kidney lesions featured extensive edema of renal tubular epithelium, pale and vacuolated cytoplasm, and necrosis and sloughing of tubular epithelial cells (Fig. 6). In summary, the IBV strains induced pathological lesions in the respiratory and urinary systems, with SX/2409 causing more severe lesions than its parental strain SX/2408.

### Horizontal transmission capability of the IBV strains

**Fig. 7:**
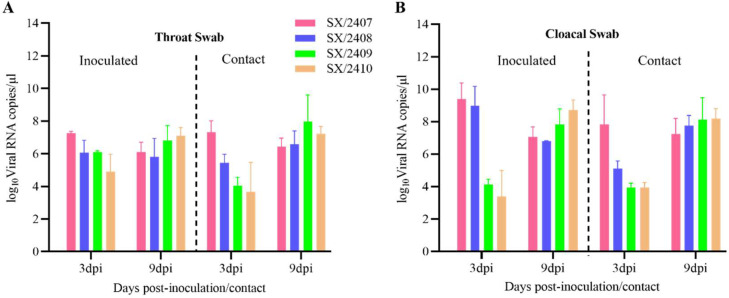
**Horizontal transmission capacity of the four IBV isolates in chickens.** (A) Viral shedding from the oropharynx of directly inoculated chickens and their contact-exposed counterparts was measured at 3 and 9 days post-infection. (B) Viral shedding from the cloaca of directly inoculated chickens and their contact-exposed counterparts was measured at 3 and 9 days post-infection.

To study the transmission dynamics within a flock, three healthy one-day-old SPF chicks were introduced as contact birds into each group at 1 dpi. Oropharyngeal and cloacal swabs were collected from both inoculated and contact birds, and viral shedding was quantified by RT-qPCR. Results showed that virus was detected in swabs from all inoculated birds (Fig. 7). Notably, high levels of viral shedding were also detected in oropharyngeal and cloacal swabs from contact birds as early as 3 dpi, and shedding persisted at least until 9 dpi (Fig. 7), indicating strong horizontal transmission capability for all four IBV strains.

### Replication of IBV isolates in chicken tissues

**Fig. 8:**
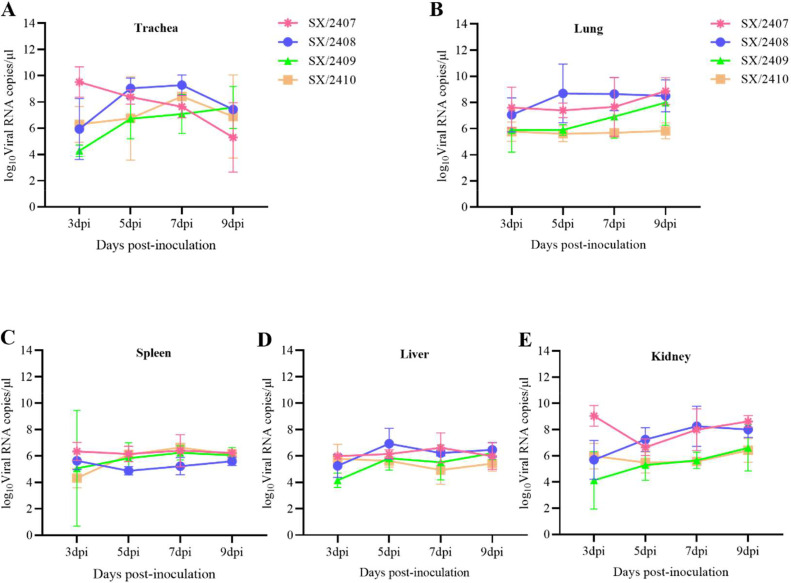
**Viral replication in various organs of SPF chickens following infection with the four IBV isolates.** At 3, 5, 7, and 9 days post-infection, three infected chickens were randomly selected from each group. Samples of the trachea (A), lung (B), spleen (C), kidney (D), and liver (E) were collected. Tissues were homogenized, and nucleic acids were extracted to determine the viral load in each organ via RT-qPCR.

To investigate viral replication in various organs, three chicks from each group were euthanized at 3, 5, 7, and 9 dpi. Tissues including trachea, lung, liver, spleen, and kidney were collected, and viral RNA loads were determined by RT-qPCR. In chicks infected with SX/2407, tracheal viral loads peaked at 3 dpi, which were significantly higher than those of the other three strains, and then gradually declined (Fig. 8A). For chicks infected with SX/2408 and SX/2410, tracheal viral loads peaked at 7 dpi and gradually decreased by 9 dpi. In contrast, tracheal viral loads in the SX/2409-infected group showed a gradual increase from 3 dpi to 9 dpi (Fig. 8A). In the lungs, viral loads in SX/2408-infected chicks peaked at 5 dpi, followed by a gradual decline at 7 and 9 dpi. For SX/2407 and SX/2409, lung viral loads increased continuously until 9 dpi. Lung viral loads in SX/2410-infected chicks remained stable at 10⁶ copies/μL from 3 to 9 dpi (Fig. 8B). Viral loads in the liver and spleen did not show a significant increase from 3 dpi, remaining at low replication levels throughout the observation period (3 to 9 dpi) (Figs. 8C and 8D). In the kidneys, viral loads in SX/2407-infected chicks peaked at 3 dpi, decreased slightly at 5 dpi, and then gradually increased at 7 and 9 dpi (Fig. 8E). For the SX/2408-infected group, kidney viral loads peaked at 7 dpi and began to decline at 9 dpi. At 3 dpi, SX/2410-infected chicks exhibited higher kidney viral loads than SX/2409-infected chicks; subsequently, both groups showed similar viral replication titers at 5 and 7 dpi, with both reaching peak levels at 9 dpi. Collectively, these results demonstrate that the four IBV isolates exhibited significantly higher viral replication titers in the trachea, lungs, and kidneys, indicating a propensity to cause more severe pathological damage to the respiratory and urinary systems.

## Discussion

IBV is one of the most significant pathogens affecting poultry health and is prevalent worldwide. The primary strategy for IBV prevention is vaccination. However, the error-prone replication mechanism of coronaviruses, combined with the complex environment of intensive farms, increases the likelihood of IBV genome mutation and recombination (Gao, et al., 2016; Lu, et al., 2024; Wu, et al., 2019). To adapt to host immune pressure from vaccination, the S1 subunit of the coronavirus often undergoes mutation or recombination to evade immune surveillance, complicating IBV control. In this study, we isolated four IBV strains from a large-scale layer farm in Changzhi, Shanxi, which practiced routine vaccination with attenuated live vaccines (4/91, LDT3-A). During virus purification, we observed dwarfism in embryos inoculated with all four strains. Phylogenetic analysis based on the S1 gene classified SX/2407, SX/2408, SX/2409, and SX/2410 into the GI-7, GI-19, GVII-1, and GVI-1 lineages, respectively. The considerable genetic distance between these four strains highlights the complexity of current IBV epidemiology when multiple genotypes co-circulate within a single farm. As documented in previous studies, GI-19 is the predominantly circulating genotype in China, with a detection rate of 35.06% in southern regions, followed by GVI-1 (33.77%), GI-7 (9.09%), and GVII-1 (2.06%) (Wang, et al., 2025). The prevalence of GI-19 strains has caused significant losses to the Chinese poultry industry in recent years.

Whole-genome recombination analysis revealed that recombination regions in SX/2407, SX/2409, and SX/2410 were predominantly located within the S gene region. SX/2407 resulted from recombination between GVI-1 and GI-7 lineages in the S gene, with an additional fragment from the LDT3-A vaccine strain. The GVII-1 strain SX/2409 and the GVI-1 strain SX/2410 both originated from recombination between the GI-19 strain SX/2408 and vaccine strains. SX/2407 and SX/2410 exhibited four recombination breakpoints, while SX/2409 had two. Based on existing reports, the presence of multiple breakpoints likely indicates sequential or complex recombination events (Li, et al., 2021; Yuan, et al., 2022). This suggests that IBV evolution in nature is not a simple linear process but rather a dynamic, network-like phenomenon. Zeng Z et al. reported that GI-19 strains recombined with the 4/91 vaccine strain exhibited multi-tissue tropism in viral replication, with a mortality rate as high as 60% (Zeng, et al., 2025). Franzo G et al. conducted molecular epidemiological analysis of GI-19 sequences collected in Spain from 2008 to 2025 and identified six distinct recombination events, some of which were clearly derived from recombination with vaccine strains (Franzo, et al., 2025). In addition to the commonalities identified in their studies, we observed that GI-19 strains can transition to other genotypes through recombination with vaccine strains, which reduces cross-protection between strains and thus enables them to acquire immune evasion capabilities (Vigil, et al., 2018). Zhou et al. isolated recombinant field strains from vaccinated flocks and found their genomes originated from multiple fragment recombination events between field and vaccine strains (Zhou, et al., 2017). The 4/91 vaccine strain demonstrated a high recombination rate, whether as a major or minor parent. Previous studies have indicated that the 4/91 vaccine can promote recombination with other strains, which is consistent with our findings (Chen, et al., 2024; Fan, et al., 2022; Feng, et al., 2018). Krisztina et al., analyzing IBV field strains of European origin, concluded that most recombination events occurred between field strains and between field and vaccine strains, with vaccine strains themselves also undergoing recombination (Bali, et al., 2021). These results align with ours, indicating that the widespread use of commercial vaccines plays an important role in generating new IBV genotypes and variants.

IBV strains are categorized based on their primary target organs and clinical manifestations into respiratory, nephropathic, proventriculitis, and reproductive types (Dai, et al., 2022). Pathogenicity trial results showed that clinical signs appeared at 3 dpi in all infected groups, and all four strains caused lesions in both the respiratory and urinary systems. Necropsy revealed kidney swelling and urate deposition in SX/2407, SX/2408, and SX/2409 infected birds. Yellowish mucus was observed in the trachea of SX/2407 and SX/2409 infected birds. Morbidity was 100%, with mortality rates of 50% (SX/2407), 20% (SX/2408), and 60% (SX/2409). Notably, SX/2409 is a recombinant strain derived from SX/2408 and a vaccine strain, and the mortality rate induced by SX/2409 was higher than that caused by SX/2408. Collectively, these findings suggest that recombination between GI-19 genotype infectious bronchitis virus (IBV) and vaccine strains may drive the evolutionary transition of IBV toward a phenotype with enhanced virulence. Tracheal ciliary activity was assessed at 3, 5, 7, and 9 days post-infection (dpi). The results revealed that all four IBV isolates exerted a significant inhibitory effect on tracheal ciliary motility and caused severe epithelial damage from 5 dpi onwards. Quantification of viral loads in visceral organs further verified that all four IBV strains were able to replicate extensively in the respiratory system. Specifically, the recombinant SX/2409 not only induced more severe renal lesions but also retained robust respiratory pathogenicity. Consequently, these IBV strains may predispose chickens to secondary infections by low pathogenic avian influenza virus (LPAIV), pathogenic *E. coli*, or other pathogens (Yang, et al., 2024).

Our findings provide new evidence supporting the notion that continuous recombination between field and vaccine strains can enhance virulence and drive viral evolution. Continuous epidemiological surveillance of IBV is necessary to promptly identify new variant strains, providing a basis for rational vaccine strain selection and early warning for potentially highly pathogenic recombinant strains. Furthermore, these results suggest caution regarding the use of multiple live vaccines of different genotypes in large-scale farms. There is an urgent need to develop novel vaccines or recombinant live vaccines based on a common genetic background (e.g., H120) to avoid complex recombination events resulting from the widespread application of diverse live vaccines. Meanwhile, according to the IBV epidemiological profile of individual farms and the local region, the use of a single matched-genotype live vaccine for immunization may be more effective. Such an approach can effectively mitigate the risk of novel recombinant viruses emerging from the concurrent use of multiple live vaccines belonging to distinct genotypes. To prevent IBV from further evolving toward increased virulence, future vaccine development should focus on novel vaccines that are recombination-deficient or recombination-disadvantageous, thereby reducing the fitness of recombinant viruses (Keep, et al., 2025).

## Conclusion

This study isolated and characterized four novel IBV strains (SX/2407, SX/2408, SX/2409, SX/2410) from a vaccinated layer farm. Whole-genome recombination analysis revealed that three of these strains (SX/2407, SX/2409, SX/2410) were recombinants involving field strains and vaccines. Animal challenge experiments demonstrated that all four strains replicated efficiently in chickens. Notably, the recombinant strain SX/2409 exhibited significantly higher pathogenicity than its major parental strain SX/2408, with a mortality rate reaching 60%. These findings provide crucial evidence that natural recombination with vaccine strains can drive the evolution of more virulent IBV phenotypes. This underscores the importance of continuous molecular surveillance and highlights the need to evaluate current vaccine immunization strategies and develop novel, safer vaccines.

## Data availability

The data that supports this study is available in the article and accompanying online supplementary material.

## CRediT authorship contribution statement

**Zhonglin Huang:** Writing – review & editing, Writing – original draft, Visualization, Validation, Supervision, Resources, Methodology, Investigation, Formal analysis, Data curation, Conceptualization. **Xu Wang:** Writing – review & editing, Visualization, Validation, Supervision, Software, Resources, Methodology, Investigation, Formal analysis, Data curation. **Hongfeng Ren:** Writing – review & editing, Validation, Supervision, Software, Methodology, Formal analysis, Data curation. **Pei Li:** Visualization, Validation, Software, Resources, Methodology, Investigation. **Yang Wang:** Software, Investigation, Formal analysis, Conceptualization. **Yafan Zhao:** Supervision, Software, Methodology, Investigation. **Jinchi Guo:** Validation, Supervision, Formal analysis. **Yingjie Chen:** Supervision, Software. **Pengran Wang:** Supervision, Investigation. **Xiaoran Li:** Supervision, Software. **Yanna Guo:** Supervision, Software, Investigation. **Jianhui Li:** Supervision, Software, Investigation. **Libin Liang:** Writing – review & editing, Writing – original draft, Visualization, Funding acquisition, Conceptualization. **Junping Li:** Writing – review & editing, Writing – original draft, Visualization, Supervision, Software, Resources, Funding acquisition, Conceptualization.

## Disclosures

We confirmed that this manuscript has not been published elsewhere and is not under consideration by another journal. All authors have approved the manuscript, agree with submission to Poultry Science and declare no conflict of interest.

## Appendix Supplementary materials

Image, application 1

## Footnotes

Supplementary material associated with this article can be found, in the online version, at doi:10.1016/j.psj.2026.106747.
