# Supplementary material for: Recombination with a vaccine strain drives the evolution of a more virulent phenotype in avian infectious bronchitis virus
Source: Poult Sci. 2026 Mar 8;105(6):106747. doi: 10.1016/j.psj.2026.106747 (PMC13068550; doi:10.1016/j.psj.2026.106747)
Supplement: Supplementary file 1 [file mmc1.docx]

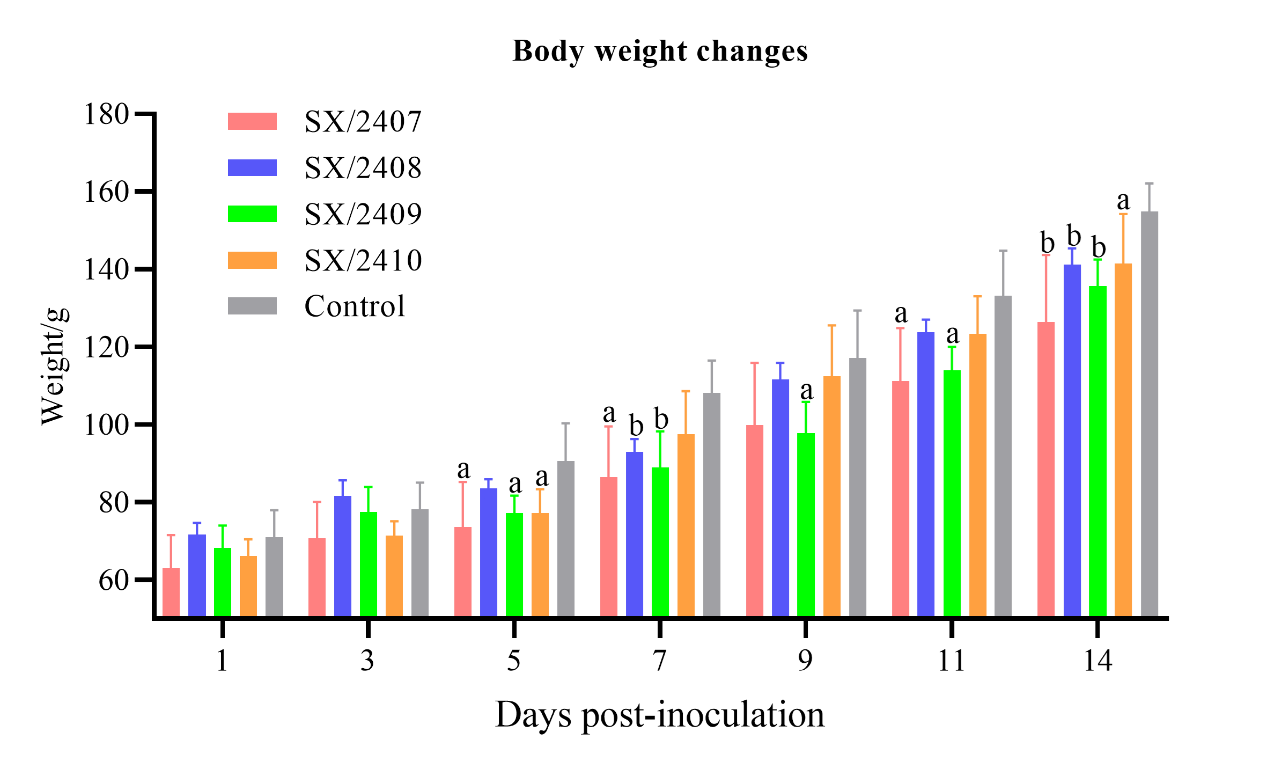


**Supplementary Fig. 1** **Body-Weight Changes in Chickens Infected with the Four IBV Strains.** Body‑weight trajectories for chickens infected with SX/2407, SX/2408, SX/2409, and SX/2410 versus PBS‑inoculated controls. Bars indicated mean ± standard deviation. Clinical scores were analyzed using Student’s t‑test, displaying only the comparisons between each infection group and the control; no significant differences were observed among the infection groups. Significance levels: a, p < 0.05; b, p < 0.01; c, p < 0.001.
